# Herbal formula SC-E1 suppresses lipopolysaccharide-stimulated inflammatory responses through activation of Nrf2/HO-1 signaling pathway in RAW 264.7 macrophages

**DOI:** 10.1186/s12906-017-1874-1

**Published:** 2017-07-28

**Authors:** Ju-Yeon Park, Young-Won Kwon, Soo Chil Lee, Sun-Dong Park, Ju-Hee Lee

**Affiliations:** College of Korean Medicine, Dongguk University, Goyang, 10326 Republic of Korea

**Keywords:** Herbal formula, RAW 264.7 macrophage, Anti-inflammatory, Mitogen-activated protein kinase (MAPK), Nuclear factor-kappa B (NF-κB), NF-E2-related factor 2 (Nrf2)

## Abstract

**Background:**

SC-E1 is a novel herbal formula consisting of five oriental medicinal herbs used frequently in traditional herbal medicine for the treatment of inflammatory diseases in Korea. This study examined the effects of SC-E1 on lipopolysaccharide (LPS)-stimulated macrophages and the molecular mechanism involved.

**Methods:**

The cytotoxic effect of the SC-E1 extract was evaluated in RAW 264.7 cells by MTT assay. The effects of SC-E1 on the free radical scavenging and generation of intracellular reactive oxygen species were measured using DPPH and DCFH-DA, respectively. The effects of SC-E1 on the production of pro-inflammatory cytokines, inflammatory mediators, and related products were determined by ELISA and western blotting. The molecular mechanism and the nuclear translocation of nuclear factor-kappa B (NF-κB) and NF-E2-related factor 2 (Nrf2) were examined by western blot analysis and immunocytochemistry.

**Results:**

SC-E1 exhibited strong anti-oxidant activity and inhibited LPS-induced NO secretion as well as iNOS expression and the production of pro-inflammatory cytokines, without affecting the cell viability. SC-E1 also suppressed the LPS-induced NF-κB activation and the mitogen-activated protein kinase (MAPK) pathway. Moreover, SC-E1 induced heme oxygenase-1 (HO-1) expression via the nuclear translocation of Nrf2. The inhibitory effects of SC-E1 on the production of pro-inflammatory cytokines were abrogated by treatment with SnPP, an HO-1 inhibitor.

**Conclusion:**

These results suggest that SC-E1 exerts its anti-oxidant and anti-inflammatory effects through the inhibition of NF-κB and MAPK as well as Nrf2-mediated HO-1 induction in macrophages. These findings provide evidences for SC-E1 to be considered as a new prescription for treating inflammatory diseases.

## Background

Inflammation is a complex immune response that protects the body from infections or tissue injury and involves the activation of various immune cells, such as macrophages, neutrophils, and lymphocytes [1]. Chronic inflammation is closely associated with the pathogenesis of many human diseases, such as rheumatoid arthritis, atherosclerosis, cardiovascular disease, sepsis, and cancer [2]. Therefore, it is very important to control inflammation to prevent progression to severe or chronic disease. Macrophages play important roles in regulating inflammation and various immune responses in the host immune system [3]. Exposure to inflammatory stimuli, such as lipopolysaccharides (LPS), which is an endotoxin from gram-negative bacteria, causes the activation of macrophages. Activated macrophages secrete inflammatory mediators, such as nitric oxide (NO) and prostaglandin E_2_ (PGE_2_), and produce inflammatory cytokines, including tumor necrosis factor (TNF)-α, interleukin (IL)-1, IL-6, and IL-10 [4, 5]. NO and PGE_2_ are synthesized by inducible nitric oxide synthase (iNOS) and cyclooxygenase-2 (COX-2), respectively [6, 7]. Although these inflammatory mediators and cytokines are essential for host survival after an infection as well as for the repair of tissue damage, their excessive production is associated with a range of inflammatory disorders [5, 8].

Oxidative stress is an imbalance between the production reactive oxygen species (ROS, free radicals) and the anti-oxidant defenses caused by excessive amounts of ROS in the body. In oxidative stress circumstances, the cells in living organisms undergo DNA fragmentation, lipid peroxidation, and protein decomposition, which cause not only aging process, but also chronic or acute disorders such as inflammation, cancer, and immune disease [9]. Therefore, many people often consume exogenous anti-oxidants from vitamins, dietary supplements, or herbal remedies to support the endogenous antioxidative defense. According to this consumption trend, many researchers have assessed in natural herbal medicines for regulating oxidative stress and scavenging free radicals. In traditional Korean medicine, herbal medicine has been developed as compositions containing various kinds of herbs to enhance the efficacy of each herb while reducing their side effects and accommodating complex clinical situations [10]. Therefore, Korean herbal medicine may provide good candidates for anti-oxidants or anti-inflammatory agents.

Based on prescriptions describing treatments for inflammation in the Sanghanron (傷寒論) and Geumgweyoryak (金匱要略), which are classical textbooks of traditional Chinese medicine, this study designed a novel anti-inflammatory herbal formula called SC-E1. The formulation was comprised of five medicinal herbs including *Gypsum, Gardenia jasminoides*, *Glycyrrhiza uralensis*, *Pueraria lobata*, and *Platycodon grandiflorum*, which have been applied frequently in prescriptions for the treatment of the inflammation. Each of these medicinal herbs was reported to exhibit anti-oxidative and/or anti-inflammatory properties. *Gypsum* is a sulfate mineral that is used in traditional Oriental Medicine for reducing fevers and alleviating thirst and sweat, at a dose of 15–60 g per day, and is mainly composed of calcium sulfate (CaSO_4_) [11, 12]. It is recognized by U.S. Food and Drug Administration (FDA) as GRAS (Generally Recognized as Safe) additive approved for food and pharmaceutical products and widely accepted as safe food additive in many countries [13]. *Gardenia jasminoides* has been shown to possess anti-oxidative, anti-inflammatory, anti-allergic properties, and anti-cancer effects from its dichloromethane fraction [14–17]. *Glycyrrhiza uralensis* is the most widely and frequently used herb in traditional medicine, and is an essential component in most herbal prescriptions [18]. This herb has anti-allergic, anti-oxidant, anti-inflammatory, anti-carcinogenic, and hepatoprotective activities [19–22]. *Pueraria lobata* was also reported to have various pharmacological properties, such as anti-obesity, anti-hyperglycemic, and anti-inflammatory activities [23–25]. In addition, the anti-inflammatory effects of *Platycodon grandiflorum* were investigated [26–28].

In the present study, SC-E1 was formulated based on the daily dose of each herbal medicine mentioned above and evaluated the anti-inflammatory and anti-oxidative properties in LPS-stimulated RAW 264.7 macrophages. Furthermore, the underlying mechanisms possibly involved in regulating the inflammatory responses by SC-E1 were elucidated.

## Methods

### Chemicals and reagents

The LPS (*E.coli* 055:B5), Griess reagent, geniposide, puerarin, 3-(4,5-dimethylthiazol-2-yl)-2,5-diphenyl-tetrazolium bromide (MTT), and other reagents were purchased from Sigma-Aldrich (St. Louis, MO, USA). The ELISA kits for TNF-α, IL-1β, and PGE_2_ were obtained from R&D Systems (Minneapolis, MN, USA). The primary antibodies for the IκB, p-IκB, p-NF-κB, p-JNK, and p-p38 were supplied by Cell Signaling Technologies (Danvers, MA, USA). Other primary antibodies and horseradish peroxidase (HRP)-conjugated goat anti-rabbit and goat anti-mouse IgGs were obtained from Santa Cruz Biotechnology (Santa Cruz, CA, USA).

### Preparation of herbal formula SC-E1 extract

SC-E1 is composed of five Oriental medicinal herbs: *Gypsum* (voucher specimen number: DUMCKM2015–041), *Gardenia jasminoides* (Rubiaceae, Fruits, voucher specimen number: DUMCKM2015–069), *Glycyrrhiza uralensis* (Leguminosae, Roots, voucher specimen number: DUMCKM2015–003), *Pueraria lobata* (Leguminosae, Roots, voucher specimen number: DUMCKM2015–001), and *Platycodon grandiflorum* (Campanulaceae, Roots, voucher specimen number: DUMCKM2015–010). All herbal medicines were purchased as dried herbs from Omniherb (Daegu, Korea) certified by the Korea Food and Drug Administration (KFDA) and authenticated by Professor Sun-Dong Park at the Department of Prescriptions, College of Korean Medicine, Dongguk University, where voucher specimens were deposited.

A mixture of *Gypsum*, *Gardenia jasminoides*, *Glycyrrhiza uralensis*, *Pueraria lobata*, and *Platycodon grandiflorum* at 16:6:2:6:3 ratios was macerated with 800 ml of 70% ethanol, stirred for 24 h at room temperature (RT), and filtered twice using 8 μm pore size Whatman filter paper. After rotary evaporation at 40 ~ 45 °C, the concentrate was lyophilized, yielding 15.9 g of dried power (yield ratio 15.9%).

### High-performance liquid chromatography (HPLC) analysis of SC-E1

Compositional analysis of the SC-E1 extract was performed using a Dionex Ultimate 3000 HPLC system (Thermo Fisher Scientific, Waltham, MA, USA) equipped with a binary solvent delivery pump, a vacuum degasser, an autosampler, a thermostated column oven, a diode array spectrophotometric detector (DAD), and Chromeleon 6.8 software. Puerarin and geniposide (Sigma-Aldrich) were used as standards. The separations were achieved on a VDSpher EC-C18 column (4.6 × 250 mm, 5 μm, VDSoptilab, Germany). The mobile phase consisted of 0.3% trifluoroacetic acid (A) and acetonitrile (B), and gradient elution was performed as follows: 10% B for 0–1 min, 10–50% B for 1–25 min, 90% B for 25–35 min, and 90–10% B for 35–40 min. The column temperature was maintained at 30 °C, and the flow rate and injection volume were 0.8 ml/min, and 10 μl, respectively. All data were acquired and processed using Chromeleon 6.8 software.

### Cell culture

RAW 264.7 cells were maintained in Dulbecco’s Modified Eagle’s Medium (DMEM) with 10% heat-inactivated fetal bovine serum (FBS) and 1% penicillin-streptomycin, and incubated at 37 °C in a humidified atmosphere containing 95% air and 5% CO_2_. During maintenance, the cells were passaged every 3–4 days. FBS, cell culture media, penicillin/streptomycin, and all other reagents used for the cell culture studies were purchased from Hyclone (Gaithersburg, MD, USA).

### Cell viability assay

The cell viability of the RAW 264.7 macrophages upon exposure to SC-E1 was detected using an MTT assay. The RAW 264.7 cells were suspended at 4 × 10^5^ cells/ml densities on 96-well plates and incubated overnight. Different concentrations of SC-E1 in serum-free medium were treated for 24 h, and MTT solution (2 mg/ml) was added to each well for another 2 h. After removing the supernatant, the insoluble crystal formazan was fully dissolved in dimethyl sulfoxide (DMSO) using a shaker, and the absorbance of each well was measured at 540 nm using a microplate reader (Genios, Tecan, Austria).

### DPPH scavenging assay and reactive oxygen species (ROS)

A DPPH assay was conducted using a slight modification of the method reported by Gyamfi et al. [29]. Reduction of the DPPH free radical was measured by reading the absorbance at 540 nm after adding each reactant. Briefly, various concentrations of samples were incubated with 50 mM Tris-HCl (pH 7.4) and 0.1 mM DPPH ethanol solution for 30 min, in a dark room.

A fluorescent 2',7'-dichlorofluorescein diacetate (DCFH-DA) assay was performed to determine the intracellular ROS concentrations. Murine macrophages were seeded on a 96-well black plate at 1 × 10^5^ cells/ml, and incubated with LPS in the presence or absence of SC-E1. After removing the medium, 10 μM DCFH-DA in PBS was added to each well at 37 °C for 30 min. The fluorescence was measured at excitation and emission wavelength of 480 nm and 530 nm, respectively, using a fluorescence microplate reader (Spectra Gemini, Molecular Devices).

### Nitric oxide production assay

RAW 264.7 macrophages were seeded on a 24-well plate at a density of 4 × 10^5^ cells/ml. After the prescribed treatment, the cell–removed supernatant was reacted with an equal volume of Griess reagent (1% sulfanilamide, 0.1% N-(1-naphthyl)-ethylenediamine dihydrochloride, 2.5% phosphoric acid), and incubated in a dark room for 10 min at RT. Nitric oxide production was determined by measuring the absorbance at 540 nm with a microplate reader (Versamax, Molecular Devices).

### Enzyme-linked immunosorbent assay (ELISA)

The contents of cytokines including TNF-α, IL-1β, and PGE_2_ were determined using ELISA kits (R&D Systems, Minneapolis, MN, USA). The macrophages were seeded on a 24-well plate at 4 × 10^5^ cells/ml, and treated with different concentrations of SC-E1 in the presence or absence of LPS (1 μg/ml) for 18 h. Fresh supernatants, without freezing and thawing, were used to evaluate the level of cytokine accumulation according to the manufacturer’s instructions.

### Western blot analysis

The cells were lysed in RIPA containing phosphatase and protease inhibitor cocktail, and the protein concentrations were evaluated using a BCA protein assay kit (Thermo Scientific, IL, USA). Equal amounts of the total proteins were subjected to a 10% SDS-Polyacrylamide gel electrophoresis and transferred onto a polyvinylidene fluoride membrane. After blocking with 5% skim milk in phosphate-buffered saline (PBS) at RT for 1 h, the membranes were incubated with the primary antibodies such as iNOS (sc-650), COX-2 (sc-376861), HO-1 (sc-10789), Nrf2 (sc-722), p-IκB (#9246), IκB (#9242), p-NF-κB (#3037), Lamin B (sc-374015), β-actin (sc-47778), p-ERK1/2 (sc-7383), p-JNK (#9251), p-p38 MAPK (#9211), ERK1/2 (sc-94), JNK (sc-7345), and p38 MAPK(sc-535) at 4 °C overnight, followed by incubation with the proper secondary antibodies conjugated with horseradish peroxidase at RT for 1 h. The blots were developed using ECL prime solution (Amersham Bioscience, Buckinghamshire, UK), and visualized using a Fusion FX7 chemiluminescence imaging system (Vilber Lourmat, Marne-la-vallée, France).

### Preparation of nuclear and cytosolic fractions

The cytosol and nuclear fractions of cell pellets were obtained using NE-PER™ Nuclear and Cytoplasmic Extraction Reagents (Thermo Scientific, Rockford, IL, USA) according to the manufacturer’s instructions. Briefly, the cells were lysed in ice-cold CER-I buffer supplemented with the protease inhibitors and incubated on ice for 10 min. After adding ice-cold CER-II, the samples were centrifuged and the supernatant solution (cytoplasmic fraction) was collected. Pellets containing the nuclei were washed three times in PBS to remove any contamination from the cytosolic proteins, and the nuclear proteins were extracted in NER buffer supplemented with protease inhibitors.

### Immunofluorescence microscopy

To detect NF-κB or Nrf2 translocation, the cells were cultured directly on glass cover slips in 6-well plate with the prescribed concentrations of SC-E1 with or without LPS. Briefly, the cells were fixed with −20 °C methanol for 10 min and permeabilized in PBS containing 1% Triton X-100 for 10 min. The cells were incubated with either NF-κB or Nrf2 antibody (1:200) in PBS overnight at 4 °C, followed by fluorescein isothiocyanate (FITC)-conjugated goat anti-rabbit IgG labelling (1:1000, Invitrogen, Carlsbad, CA, USA) for 1 h and 4,6-diamidino-2-phenylindole (DAPI, Sigma-Aldrich) for 5 min. After mounting the coverslips on glass slides using ProLong® Gold Antifade Reagent (Thermo Fisher scientific, IL, USA), fluorescence images were captured using an Olympus BX50 fluorescence microscope (Olympus Optical Co., Tokyo, Japan).

### Statistical analysis

All the results are expressed as the mean ± SD. Statistical analyses were performed using one-way analysis of variance (ANOVA) followed by a Tukey’s multiple comparison. A *p* value <0.05 was considered significant. All experiments were performed independently at least 3 times, and the data were analyzed using Prism 5.0 software (GraphPad Software, Inc., San Diego, CA, USA).

## Results

### HPLC analysis of SC-E1

**Fig. 1:**
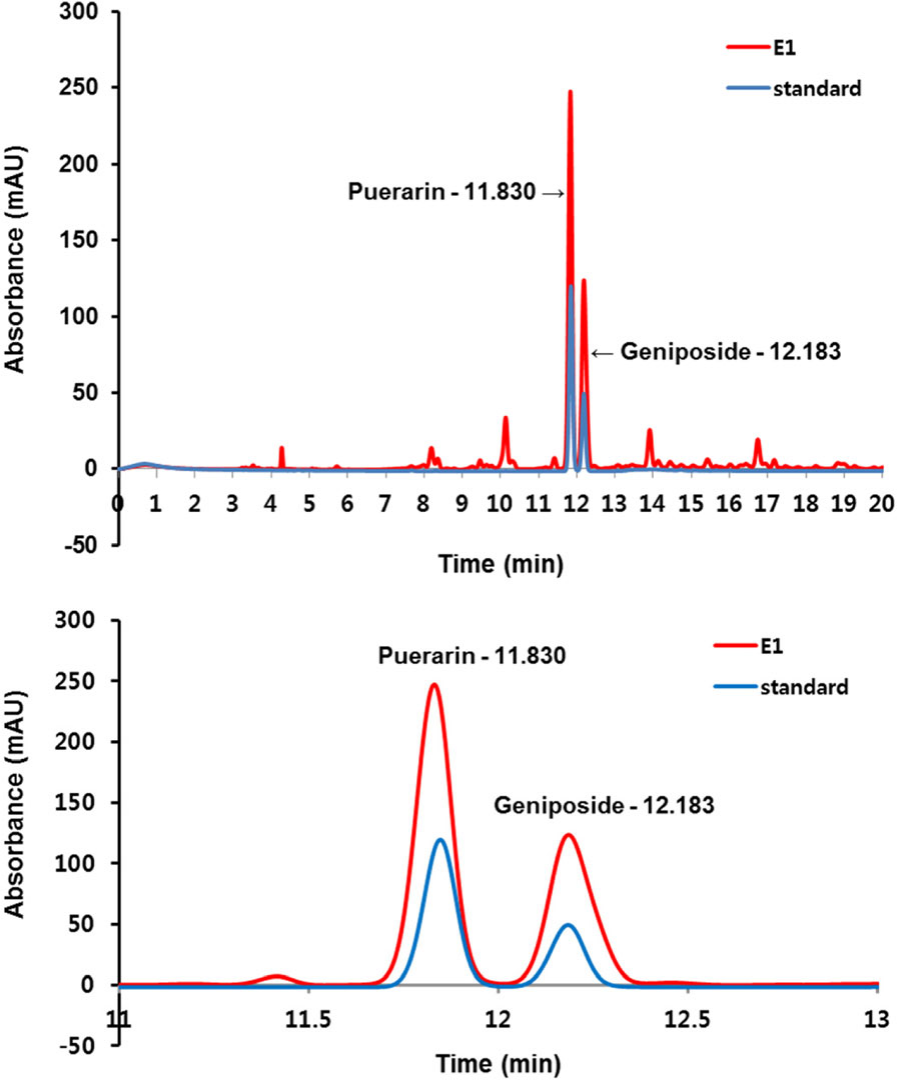
HPLC chromatograms of the SC-E1 at 240 nm

To conduct a quantitative HPLC analysis of SC-E1, the two compounds, geniposide from *Gardenia jasminoides* and puerarin from *Pueraria lobata*, were selected as the marker compounds for quality control. Figure 1 presents a typical HPLC chromatogram of SC-E1 extracts. Calibration curves for the two components showed good linearity with correlation coefficient (*r* ^2^) ≥ 0.9996 in their various concentration ranges. The contents of the two compounds were calculated from the calibration curve of the standard. The retention times of the two components, geniposide and puerarin, were 12.183 and 11.830 min, respectively. The amounts of geniposide and puerarin were 80.3 mg/kg and 45.4 mg/kg, respectively.

### Effects of SC-E1 on the oxidative stress in RAW 264.7 macrophages

**Fig. 2:**
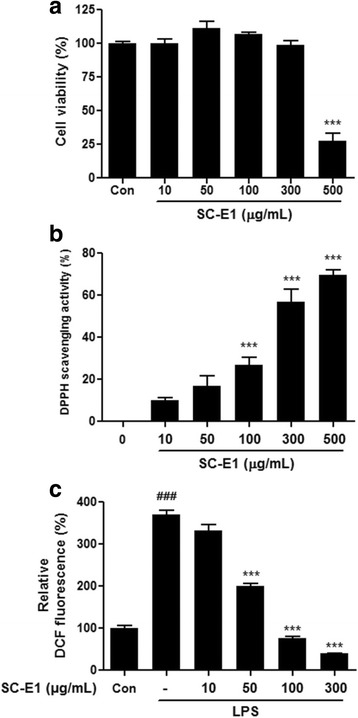
Effects of SC-E1 on the cell viability and oxidative stress. **a** Cell viability of SC-E1 treated RAW 264.7 macrophages. RAW 264.7 macrophages were treated with 10–500 μg/ml of SC-E1 for 24 h. The cell viability was measured by an MTT assay. **b** Anti-oxidant activity of SC-E1 was evaluated by a DPPH scavenging assay. Data are represented as the percent of DPPH radical inhibition. **c** Effect of SC-E1 on ROS generation. Intracellular H_2_O_2_production was monitored by measuring the DCF fluorescence using a fluorescence microplate reader

To examine the cytotoxic effects of SC-E1, the viability of RAW 264.7 macrophages was evaluated at concentrations of 10–500 μg/ml SC-E1. As shown in Fig. 2a, SC-E1 was not cytotoxic up to 300 μg/ml, but treatment with 500 μg/ml caused significant cytotoxicity. The in vitro anti-oxidant activity of SC-E1 was measured using a DPPH assay, which is commonly used to determine the free radical scavenging activity. The DPPH radical scavenging activity of SC-E1 was very strong (Fig. 2b). In addition, the effects of SC-E1 on oxidative stress were evaluated by measuring the ROS level in LPS-stimulated RAW 264.7 macrophage cells. According to the cell viability results, the cells were treated with SC-E1 at a maximum concentration of 300 μg/ml. As expected, LPS exposure increased intracellular ROS production significantly, which was suppressed by SC-E1 treatment in a dose-dependent manner (Fig. 2c). This suggests that SC-E1 has strong anti-oxidant activity.

### Effect of SC-E1 on the production of pro-inflammatory mediators and cytokines in LPS-stimulated RAW 264.7 macrophages

**Fig. 3:**
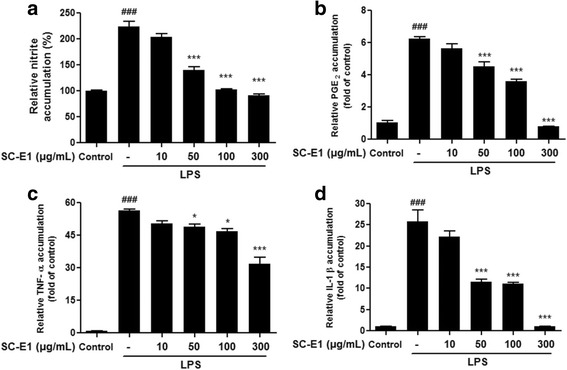
Effect of SC-E1 on the production of pro-inflammatory mediators and cytokines in LPS-stimulated RAW 264.7 macrophages. **a** Effect of SC-E1 on nitrite production in LPS-stimulated RAW 264.7 cells. The cells were stimulated with 1 μg/ml of LPS, in the absence or presence of various concentrations (10, 50, 100, 300 μg/ml) of SC-E1 for 24 h. Nitrite production by LPS treated macrophages was measured using Griess reagent. Effects of SC-E1 on the secretion of (**b**) PGE_2_, (**c**) IL-1β (**d**), and TNF-α. (Significant compared to the control, ^###^ *p* < 0.001, significant as compared to LPS alone, *p < 0.05 and ***p < 0.001)

The anti-inflammatory effect of SC-E1 was examined by measuring the production of pro-inflammatory mediators, such as NO and PGE_2_. As shown in Fig. 3a, SC-E1 dose-dependently suppressed the LPS-induced NO production. In particular, 100 μg/ml and 300 μg/ml SC-E1 inhibited NO secretion to a similar extent to the control. SC-E1 also inhibited LPS-induced PGE_2_ production in a dose-dependent manner (Fig. 3b). In addition, the effects of SC-E1 on the LPS-induced production of the pro-inflammatory cytokines, IL-1β and TNF-α, were evaluated. Compared to the control, the levels of IL-1β and TNF-α were increased markedly in the LPS-stimulated RAW 264.7 cells. Pretreatment with SC-E1 at 50, 100, 300 μg/ml suppressed this augmentation (Fig. 3c–d).

### Effect of SC-E1 on the expression of iNOS and COX-2 in LPS-stimulated RAW 264.7 macrophages

**Fig. 4:**
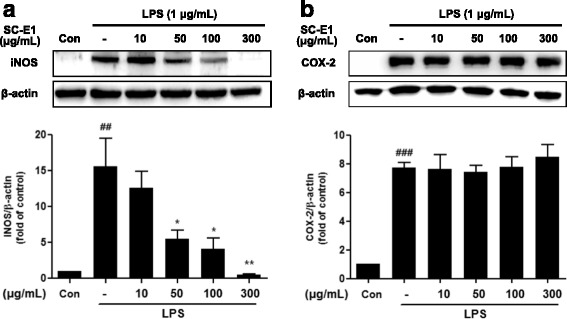
Effects of SC-E1 on the expression of iNOS and COX-2 in LPS-stimulated RAW 264.7 macrophages. Western blotting showed that the protein levels of iNOS (**a**) and COX-2 (**b**) were considerably higher in the LPS-treated RAW 264.7 macrophages than in the controls. SC-E1 pretreatment reduced the LPS-induced increases in iNOS protein levels. β-actin was used as an internal control. Each experiment conducted for 3 repeats per conditions. (Significant compared to the control, ^##^ *p* < 0.01 and ^###^ *p* < 0.001, significant compared to LPS alone, *p < 0.05 and **p < 0.01)

Because SC-E1 inhibited NO production, this study next examined whether SC-E1 modulates the expression of iNOS, an inducible enzyme that produces NO in response to LPS. As shown in Fig. 4a, SC-E1 (> 50 μg/ml) pretreatment suppressed the LPS-induced expression of iNOS in a dose-dependent manner. In particular, 300 μg/ml SC-E1 completely prevented these changes by LPS.

To investigate whether the inhibitory effect of SC-E1 on PGE_2_ production is related to the expression of COX-2, the expression of COX-2 in LPS-stimulated macrophages was determined by western blot. As shown in Fig. 4b, the expression of COX-2 was increased significantly by LPS stimulation, but the SC-E1 pretreatment had no effect on the induction of COX-2 by LPS.

### Effect of SC-E1 on MAPK signaling pathway in LPS-stimulated RAW 264.7 macrophages

**Fig. 5:**
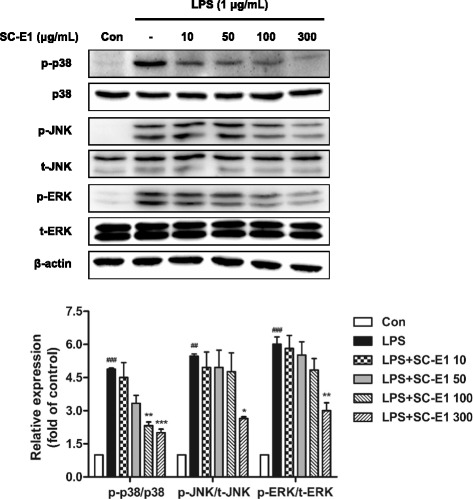
Effects of SC-E1 on the MAPK pathway activation in LPS-stimulated RAW 264.7 macrophages. The activation of the ERK, JNK, and p38 protein was detected by immunoblotting. Each experiment conducted for 3 repeats per conditions. (Significant compared to the control, ^##^ *p* < 0.01 and ^###^ *p* < 0.001, significant compared to LPS alone, *p < 0.05, **p < 0.01, and ***p < 0.001)

To reveal the mechanisms responsible for anti-inflammatory effects of SC-E1, we determined whether SC-E1 affected on the mitogen-activated protein kinases (MAPKs), LPS-mediated signaling pathway. The phosphorylation of MAPK family members p38 MAPK, JNK, and ERK reached their maximum at 30 min of LPS stimulation compared to the control (data not shown). Therefore, the cells were pretreated with SC-E1 for 18 h and stimulated with LPS for 30 min. As shown in Fig. 5, treatment with SC-E1 (10–300 μg/ml) suppressed the LPS-induced phosphorylation of p-38 MAPK, without altering its total protein levels. In addition, the expression of phosphorylated JNK and ERK were also inhibited by SC-E1, without affecting their total protein level (100 or 300 μg/ml).

### Effect of SC-E1 on NF-κB activation in LPS-stimulated RAW 264.7 macrophages

**Fig. 6:**
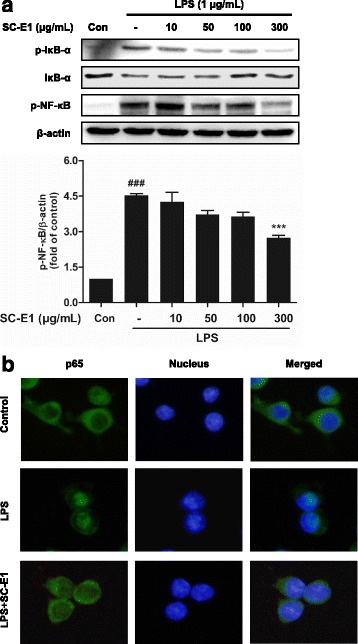
Effects of SC-E1 on the NF-κB activation in LPS-stimulated RAW 264.7 macrophages. **a** Western blotting showed the protein level of p-NF-κB p65 was increased by treating naïve cells with LPS, but that SC-E1 pretreatment inhibited this LPS-induced increase. β-actin was used as a loading control. Each experiment conducted for 3 repeats per conditions. (Significant compared to the control, ^###^ *p* < 0.001, significant compared to LPS alone, ***p < 0.001.) **b** NF-κB activation was assessed immunocytochemically. The image shows the nuclear translocation of NF-κB p65 in LPS-treated RAW 264.7 macrophages compared to the controls. SC-E1 (300 μg/ml) pretreatment inhibited the LPS-induced nuclear translocation of NF-κB p65

To further determine the mechanism underlying the anti-inflammatory effects of SC-E1, this study examined whether SC-E1 controls the activation of NF-κB signaling, relating to regulation of the expression of inflammatory mediators and cytokines [30]. The phosphorylation and degradation of IκB-α, which inhibits the activation of NF-κB, in SC-E1 pretreated macrophages under LPS-induced inflammatory conditions were first assessed by western blot analysis. The protein level of phosphorylated IκB-α increased after 1 h of LPS stimulation, whereas that of total IκB-α decreased; this change was inhibited in response to the SC-E1 pretreatment (Fig. 6a).

Next, the translocation of NF-κB in macrophages under the same conditions was examined by western blot and immunostaining. NF-κB was phosphorylated after treatment with LPS, which was prevented by pretreatment with SC-E1 (50–300 μg/ml). Moreover, the fluorescence image showed that 300 μg/ml SC-E1 suppresses the translocation of NF-κB p65 into the nucleus in macrophages despite the LPS-induced inflammatory condition (Fig. 6b).

### Effect of SC-E1 on Nrf2/HO-1 signaling in RAW 264.7 macrophages

**Fig. 7:**
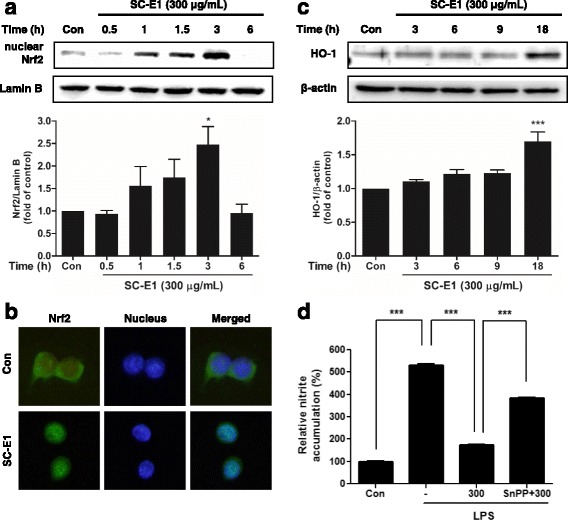
Effects of SC-E1 on the Nrf2 activation in RAW 264.7 macrophages. **a** Nuclear accumulation of Nrf2. Nrf2 was immunoblotted in the nuclear fractions of the cells treated with 300 μg/ml of SC-E1 for 0.5–6 h. Immunoblotting for Lamin B verified the equal loading of nuclear proteins. Each experiment conducted for 3 repeats per conditions. (Significant compared to the control, *p < 0.05 and ***p < 0.001.) **b** Immunofluorescence. RAW 264.7 cells were treated with 300 μg/ml of SC-E1 for 3 h. The staining of Nrf2 and DAPI was conducted as described in the method section. **c** Immunoblotting for HO-1. The HO-1 was immunoblotted in the RAW 264.7 cells that had been treated with 10–300 μg/ml of SC-E1 for 3 h. **d** Inhibitory effects of SC-E1 pretreatment and co-pretreatments with SC-E1 and SnPP (a HO-1 inhibitor) on the LPS-induced production of nitrite. RAW 264.7 cells were pretreated with SC-E1 (μg/ml) for 1 h in the presence or absence of SnPP (50 nM, 30 min), and then stimulated with LPS (1 μg/ml) for 24 h

Because SC-E1 showed potential anti-oxidant activity, this study examined whether SC-E1 was associated with Nrf2/HO-1 signaling in macrophages. The expression of Nrf2 in RAW 264.7 cells was induced by treatment with 300 μg/ml SC-E1 from 1 h to 3 h (Fig. 7a). As shown in Fig. 7b, treatment with 300 μg/ml SC-E1 for 3 h resulted in the nuclear translocation of Nrf2.

Because Nrf2 translocation is correlated with the expression of anti-oxidant proteins protecting against inflammation-induced oxidative damage, this study examined whether SC-E1 regulates HO-1, a target gene of Nrf2, in RAW 264.7 cells. The result showed that SC-E1 began to increase the HO-1 protein levels from 18 h (Fig. 7c). In addition, to investigate whether the anti-inflammatory effect of SC-E1 was mediated by HO-1 induction, Snpp, a well-known HO-1 inhibitor, was used to block HO-1 expression. As expected, the inhibition of HO-1 induction by SnPP leads to an increase in the nitrite level in SC-E1 pretreated macrophages under LPS-induced inflammatory conditions (Fig. 7d). These results suggest that pretreatment with SC-E1 exhibits anti-oxidant and anti-inflammatory effects through the induction of HO-1 via Nrf2 translocation.

## Discussion

Systemic or chronic inflammation may be the root cause of most diseases including cancers, degenerative diseases, and obesity, and can make people more susceptible to aging and disease. Although nonsteroidal anti-inflammatory drugs (NSAIDs, i.e., aspirin, ibuprofen, and naproxen) are commonly used to treat inflammatory disorders, the long-term use of these drugs can lead to undesirable side effects such as gastrointestinal bleeding and renal failure [31–33]. Recently, increasing numbers of people are choosing natural herbs or complementary and alternative medicines to prevent or relieve inflammation. Because traditional Korean herbal medicines are used as complementary and alternative medicines for a range of diseases, they may provide good natural sources for new drug discovery. This study evaluated the anti-oxidant and anti-inflammatory activities of SC-E1, a novel formulation designed for inflammation treatment by reviewing the traditional Korean medicine literature. Furthermore, the underlying mechanisms with a focus on MAPKs, NF-κB, and Nrf2 were examined.

Macrophages play important roles in the regulation of inflammation and immune responses [3]. Under LPS exposure conditions, macrophages activate and promote the productions of inflammatory mediators and cytokines, and the activation of inflammatory signaling [5]. These results showed that SC-E1 treatment suppressed the LPS-induced production of NO, upregulation of iNOS expression, and released the pro-inflammatory cytokines, such as IL-1β and TNF-α. Interestingly, the production of PGE_2_ induced by LPS was suppressed by the SC-E1 treatment without affecting COX-2 expression. The levels of PGE_2_ are regulated by the balance between COX-2 driven synthesis and 15-hydroxyprostaglandin dehydrogenase (15-PGDH)-mediated degradation of PGE_2_ [34, 35]. 15-PGDH, an enzyme degrading prostaglandin, metabolizes active PGE_2_ to biologically inactive 15-ketoprostagladins [36]. Recent studies have shown that 15-PGDH plays a key role in cancer progression and its inhibition is associated with the enhancement of tumor cell proliferation [36–39]. Considering this knowledge, SC-E1 might suppress LPS-induced PGE_2_ by up-regulating 15-PGDH independently of COX-2. Further study will be needed to verify this.

LPS activates the receptors by binding Toll-like receptor 4 and triggers the MAPK pathway, a major intracellular signaling pathway, and once activated, this pathway leads to the activation of NF-κB [40]. Therefore, the MAPK pathways have been considered molecular targets for anti-inflammatory therapy in the past few decades [41]. The increased activity of MAPK, in particular p38 MAPK, involves the production and activation of inflammation mediators and cytokines [41]. Our results showed that SC-E1 inhibits the phosphorylation of MAPKs. In particular, p38 MAPK and ERK are regulated more than JNK. Because NF-κB plays a key role in regulating the pro-inflammatory mediators and it is modulated by the MAPK pathway [42], we were also trying to elucidate the mechanism underlying inhibition of pro-inflammatory cytokines and mediators. The translocation of NF-κB into the nucleus plays a key role in the gene regulation and transcription of immune mediators [43]. Our data indicate that SC-E1 blocks the nuclear translocation of NF-κB and IκB-α degradation in RAW 264.7 macrophages under inflammation conditions. In addition, SC- E1 suppressed the LPS-induced release of pro-inflammatory cytokine TNF-α and IL-1β. These findings suggest that SC-E1 has anti-inflammatory effects by inhibiting the MAPK/NF-κB signaling pathway.

Recent studies have indicated Nrf2, as an anti-oxidant gene regulator, encoding phase II detoxifying enzymes, and this signaling pathway has critical importance in the mechanism of cellular protection and maintenance [44, 45]. HO-1 induction by activated Nrf2 protects the cells against oxidative stress [46]. SC-E1 has strong anti-oxidant activity via its free radical scavenging ability and ability to reduce the intracellular ROS level in RAW 264.7 macrophages. Therefore, we determined whether the anti-oxidant activity of SC-E1 was mediated by Nrf2/HO-1 signaling. As expected, Nrf2 nuclear translocation from the cytoplasm was potentiated and reached the peak expression in 3 h after the SC-E1 treatment. In addition, SC-E1 treatment increased the HO-1 protein expression levels in RAW 264.7 macrophages. Ashino et al. reported that enhanced HO-1 production may result in the reduction of iNOS expression and decrease the amount of free radicals [47]. In addition, Oryeongsan and Gyeji-tang, which are traditional herbal prescriptions, have been reported to have anti-inflammatory effects through the induction of HO-1 in macrophage cells [48, 49]. Consistent with these studies, the present data showed that the inhibition of HO-1 induction by SnPP leads to an increase in the nitrite levels of SC-E1 pretreated macrophages under LPS-induced inflammatory conditions. These results suggest that SC-E1 has an anti-oxidant and anti-inflammatory effect through the Nrf2/HO-1 signaling pathway.

## Conclusions

The present study showed that a novel herbal formula SC-E1 has strong anti-oxidant activity and inhibits LPS-induced pro-inflammatory mediators and cytokines. The results suggest that the beneficial effects of SC-E1 are associated with the inhibition of NF-κB and MAPK as well as Nrf2-mediated HO-1 induction in macrophages. Overall, SC-E1 could be recommended as a new prescription for treating inflammatory diseases. To further confirm these findings, various preclinical studies will be needed to investigate the toxicological and anti-inflammatory effects of SC-E1.

## Abbreviations

15-PGDH: 15-hydroxyprostaglandin dehydrogenase
ANOVA: analysis of variance
COX-2: cyclooxygenase-2
DAPI: 4,6-diamidino-2-phenylindole
DCFH-DA: 2',7'-Dichlorofluorescein diacetate
ELISA: enzyme-linked immunosorbent assay
FITC: fluorescein isothiocyanate
HO-1: heme oxygenase 1
HPLC: high-performance liquid chromatography
HRP: horseradish peroxidase
IL-1β: interleukin-1β
IL-6: interleukin-6
iNOS: inducible nitric oxide synthase
LPS: lipopolysaccharide
MAPK: mitogen-activated protein kinase
MTT: 3-(4,5-dimethylthiazol-2-yl)-2,5-diphenyl-tetrazolium bromide
NO: nitric oxide
Nrf2: Nuclear factor erythroid 2-related factor 2
NF-κB: Nuclear factor-kappa B
PBS: phosphate-buffered saline
PGE_2_: prostaglandin E_2_
ROS: reactive oxygen species
RT: room temperature
TNF-α: tumor necrosis factor-α
